# SPME-LC/MS-based serum metabolomic phenotyping for distinguishing ovarian cancer histologic subtypes: a pilot study

**DOI:** 10.1038/s41598-021-00802-9

**Published:** 2021-11-17

**Authors:** Mariola Olkowicz, Hernando Rosales-Solano, Vathany Kulasingam, Janusz Pawliszyn

**Affiliations:** 1 Department of Chemistry, University of Waterloo, Waterloo, ON N2L 3G1 Canada; 2 Department of Laboratory Medicine and Pathobiology, University of Toronto, Toronto, ON M5S 1A8 Canada; 3 Division of Clinical Biochemistry, University Health Network, Toronto, ON M5G 2C4 Canada

**Keywords:** Ovarian cancer, Cancer metabolism, Diagnostic markers, Mass spectrometry, Medical and clinical diagnostics

## Abstract

Epithelial ovarian cancer (EOC) is the most common cause of death from gynecological cancer. The outcomes of EOC are complicated, as it is often diagnosed late and comprises several heterogenous subtypes. As such, upfront treatment can be highly challenging. Although many significant advances in EOC management have been made over the past several decades, further work must be done to develop early detection tools capable of distinguishing between the various EOC subtypes. In this paper, we present a sophisticated analytical pipeline based on solid-phase microextraction (SPME) and three orthogonal LC/MS acquisition modes that facilitates the comprehensive mapping of a wide range of analytes in serum samples from patients with EOC. PLS-DA multivariate analysis of the metabolomic data was able to provide clear discrimination between all four main EOC subtypes: serous, endometrioid, clear cell, and mucinous carcinomas. The prognostic performance of discriminative metabolites and lipids was confirmed via multivariate receiver operating characteristic (ROC) analysis (AUC value > 88% with 20 features). Further pathway analysis using the top 57 dysregulated metabolic features showed distinct differences in amino acid, lipid, and steroids metabolism among the four EOC subtypes. Thus, metabolomic profiling can serve as a powerful tool for complementing histology in classifying EOC subtypes.

## Introduction

Patients with ovarian cancer (OC) often present initially with a pelvic mass of unknown malignant potential^1,2^. Each year, more than 200,000 women in North America undergo exploratory surgery for pelvic masses, with an average of 13–21% of these lesions proving to be malignant^3^. The ability to accurately discriminate between OC and benign pelvic lesions is critical for developing appropriate treatment plans, which is crucial for improving patient outcomes^1,3,4^^.^. Non-malignant lesions do not need to be treated; rather, they simply need to be monitored, thus avoiding the deleterious effects of over-diagnosis and over-treatment. Approximately 20% of women will develop an ovarian cyst or pelvic mass in their lifetime, with many undergoing unnecessary surgery as a result^3^.

Imaging, menopausal status, and serum biomarkers (including carbohydrate antigen 125 –CA125) can all be of use in distinguishing between malignant and benign pelvic masses^4,5^. As such, a number of algorithms, such as the Risk of Malignancy Index (RMI) or the Risk of Ovarian Malignancy Algorithm (ROMA)^6^, have been developed to help physicians make quick and accurate referrals. While these algorithms offer high negative predictive values (i.e., malignancy is excluded when results are negative), they are significantly limited by a lack of specificity. In particular, this lack of specificity results in a high number of false positives, which in turn results in unnecessary follow-up surgical procedures. High sensitivity is critical in ensuring that women with cancer are receiving surgical treatment from the most qualified medical personnel (i.e., gynecologic oncologists), while high specificity prevents over-diagnosis and over-treatment, thus ensuring the most efficient use of gynecologic oncologists’ time. As such, the lack of specificity in current algorithms has inhibited the efficient distribution of medical resources, led to unnecessary surgical procedures, and negatively impacted disease outcomes^6^.

The medical community’s understanding of OC has changed significantly over the past few years^4,5^. It is now evident that OC is not a single disease, but is a category comprised of several distinct histotypes. The main histotypes are epithelial in origin and include high-grade serous carcinoma (HGSeC), endometrioid carcinoma (EC), clear cell carcinoma (CCC), low-grade serous carcinoma (LGSeC), and mucinous carcinoma (MC). Epithelial histotypes differ from one another in many aspects, such as origin, response to treatment, and aggressiveness. This prior understanding of OC as a single disease rather than a number of different subtypes was a significant reason for the lack of major breakthroughs in improving OC outcomes^4^. Current algorithms are most reliable in cases of advanced HGSeC due to their emphasis on CA125^7^. Nonetheless, the management of negative test results in the presence of an apparent isolated adnexal mass remains a key challenge^8^. Indeed, reliance on current algorithms may cause medical personnel to miss an unacceptably large number of cases of early stage cancer, which is a significant oversight, as outcomes for such patients could potentially be improved if referred to a gynecologic oncologist in a timely manner. As such, it is essential to develop new methods capable of discriminating solitary adnexal masses via biochemical markers of non-serous histologic subtypes^8,9^. Identifying pathways associated with the development of non-serous OC may provide insights into disease pathogenesis and aid in the identification of biomarkers that can be used for early interventions.

Metabolites are the final products of complex metabolic pathways and most closely reflect phenotypic manifestations of disease^10^. Metabolomics seeks to identify the downstream effects of the actions of enzymes and proteins, as well as environmental exposures. Evaluating the serum profiles of patients with the various different subtypes of OC would provide snapshots of the metabolic changes linked to the changing disease phenotype^11^. In particular, nuclear magnetic resonance (NMR) spectroscopy and liquid chromatography-mass spectrometry (LC/MS) can be applied to identify metabolites in biofluids, with the aim of developing a better understanding of disease-induced processes/events and responses^10,11^. In cancer research, metabolomics-based techniques provide an impetus for the discovery of novel biomarkers and the exploration of the molecular mechanisms that underlie cancer development and progression^12,13^. Moreover, combining metabolomics with specific, sensitive sampling/extraction approaches makes it is possible to comprehensively map the metabolomic landscape.

Solid-phase microextraction (SPME) is a non-exhaustive technique that is capable of extracting a broad range of metabolites from a diversity of biological samples, including biofluids and tissues^14–16^. In SPME, a small amount of extractive phase is immobilized onto the outer part of a solid support, which is then used to extract either volatile (headspace) or non-volatile (direct immersion) analytes from a given sample matrix. Headspace-SPME-gas chromatography (GC)/MS has been widely used for the metabolic profiling of volatile compounds from complex biological samples, and has provided encouraging results, particularly in in vivo and *on-site* contexts; however, combining direct immersion (DI)-SPME with LC/MS enables the exploration of a more comprehensive range of metabolites^16^. Given the variety of available biocompatible SPME extraction phases with limited porosity, it is possible to achieve high selectivity towards small molecules, while avoiding the co-extraction of proteins and other macromolecules^14,15^. Furthermore, due to their anti-fouling properties, such SPME coatings do not induce adverse events when immersed in biological matrixes, thus making them particularly suitable for the in vivo sampling of complex matrixes^17–20^. The obtained extracts do not contain copious amounts of phospholipids, which effectively eliminates (or drastically minimizes) the matrix effects that would be encountered in LC/MS runs with extracts obtained via exhaustive extraction methods, involving solvents. The use of SPME as a sample-preparation tool in metabolomics investigations provides a number of notable advantages, including: (i) a simple workflow due to its use of coated devices with tunable extraction phases, geometries, and dimensions; (ii) the ability to consolidate several analytical steps—such as sampling, sample preparation/extraction, and metabolism quenching—into a single step; (iii) the ability to capture particularly elusive pools of metabolites (short-lived and unstable compounds); and (iv) a non-destructive nature and a minimally invasive sampling/extraction workflow, which make it especially convenient for in vivo studies. In addition, SPME features simple device design, which enables efficient workflows and makes it highly suitable for coupling with a Concept-96 autosampler for high-throughput analyses^21,22^.

In this paper, we propose a state-of-the-art analytical pipeline for comprehensive metabolic profiling in order to characterize the serum metabolite signatures of patients with various histopathological subtypes of OC. As our findings show, the proposed method is capable of providing a comprehensive picture of the metabolic differences between subsets of metabolites. Future research could build off of these findings and explore how useful these differences are in early diagnoses and interventions for the various subtypes of OC.

## Methods

### Study participants

Metabolomic alterations in serum were studied using a sample comprised of females with serous (*n* = 11), endometrioid (*n* = 10), clear cell (*n* = 10), and mucinous (*n* = 9) carcinoma. The clinicopathological characteristics of these patients are presented in Table 1.

**Table 1:** Clinicopathological data of the patients included in the study.

	Serous carcinoma *n* (%)	Endometrioid carcinoma *n* (%)	Clear cell carcinoma *n* (%)	Mucinous carcinoma *n* (%)
**Number of patients**	11 (27.5)	10 (25)	10 (25)	9 (22.5)
**Stage of disease**				
I	–	1 (10)	–	–
IA	3 (27)	3 (30)	2 (20)	2 (22)
IC	–	–	6 (60)	3 (33)
IIA	–	2 (20)	–	–
IIB	–	2 (20)	1 (10)	–
III	–	–	–	1 (11)
IIIB	1 (9.1)	–	–	–
IIIC	2 (18)	1 (10)	1 (10)	–
IV	5 (45)	–	–	2 (22)
N/A	–	1 (10)	–	1 (11)
**Malignant site**				
Ovary	11 (100)	9 (90)	10 (100)	6 (67)
Pancreas	–	–	–	1 (11)
Extrahepatic bile ducts-distal	–	–	–	1 (11)
Corpus uteri	–	1 (10)	–	–
Not primary ovarian cancer	–	–	–	1 (11)
**CA125 (U/mL)**^a ^				
I	77 (5-832)			
II	95 (19-3135)			
III	393.5 (3-1454)			
IV	947 (7-6880)			

N/A – data not available.

^a^Data expressed as median and range for a given stage of the disease.

### Materials

SPME fibers consisting of 200 µm nitinol wires with extraction phase dimensions of 7 mm × 45 µm (length × thickness) were acquired from Supelco (Merck). Two extractive phases were selected for use in this study: octadecyl-functionalized silicate particles (C18), and mixed-mode (MM – C8/SCX) particles comprised of octyl-bonded material and a strong cation exchanger. The C18-SPME fibers were used for lipidomic investigations, while the MM-SPME fibers were chosen for general metabolomic investigations.

### Extraction of analytes and instrumental analysis

Extractions were performed by immersing the fibers into aliquots containing 70 µL of sample for 60 min at 1500 rpm on an orbital shaker. Immediately after extraction, the fibers were cleaned with a Kimwipe and rinsed with purified water (MM-SPME probes) or a solution containing 10% acetone (v/v; C18-SPME probes) for 10 s to remove any trace biological material from the coating surface. Prior to instrumental analysis, the SPME fibers were desorbed in 60 µL of ACN/MeOH/H_2_O (4/4/2, v/v/v) (MM probes) or MeOH/IPA/H_2_O, 45:45:10 (v/v/v) (C18 probes) using mechanical agitation at 1500 rpm for 60 min. The extracts obtained from the desorption of the MM-SPME probes were split into two separate fractions for use in subsequent independent instrumental evaluations using two distinct chromatographic modes. In order to monitor LC/MS performance across sample runs, a quality control (QC) sample was prepared as a pooled mixture of sample aliquots and injected along the sequence.

LC/MS analysis was performed using a Vanquish UHPLC system (Thermo Scientific) interfaced with a high-resolution benchtop Exactive Orbitrap mass spectrometer (Thermo Scientific). Metabolomic investigations were conducted using Discovery HS F5 (100 × 2.1 mm, 3 µm particle size, Supelco) and ZIC-HILIC (100 × 2.1 mm, 3.5 µm, Millipore) columns. The binary mobile phase consisted of deionized water (A) and ACN (B) with either 0.1% formic acid (ESI + mode) or 1 mM acetic acid (ESI- mode). The conditions used for the chromatographic separations and relevant mass detection have been detailed in Supplementary Information. For the lipidomic investigations, chromatographic separation was achieved using an XSelect CSH C18 column (2.1 × 75 mm, 3.5 µm) using a two-solvent system combining Solvent A (40:60 MeOH:H_2_O with 10 mM ammonium acetate and 1 mM acetic acid in positive mode, and 0.02% acetic acid in negative mode) and Solvent B (90:10 IPA:MeOH with 10 mM ammonium acetate and 1 mM acetic acid in positive mode, and 0.02% acetic acid in negative mode). For a detailed description of the analytical method used in this research, please refer to Monnin et al.^23^ and see Supplementary Methods attached to this paper. Instrument control, data collection, and analysis were achieved using Thermo Xcalibur 4.0 software.

### Data processing and statistical analysis

The raw MS data were converted to mzXML files using ProteoWizard MSConvert^24^ and subsequently processed with XCMS package software for peak extraction, grouping, retention-time correction, and peak filling^25^. The IPO package was used to optimize and further adjust the XCMS parameters to be slightly more inclusive^26,27^. The extracted peaks were annotated using the xMSannotator Integrative Scoring Algorithm with HMDB, KEGG, and LIPID MAPS as the reference databases^28^. Only unique features with medium to high confidence matches annotated by HMDB/KEGG/LIPID MAPS were selected for further investigation. Principal component analysis (PCA) was performed on log-transformed Pareto-scaled data in order to detect potential outliers, assess data quality, and visualize major structures in the data. Partial least squares-discriminant analysis (PLS-DA) was applied to differentiate the metabolomic/lipidomic profile spectra of females affected by specific cancer subtypes. Potential distinguishing features were identified based on the variable importance in projection (VIP) values obtained via the PLS-DA model (variables with VIP values of ≥ 1.5 were considered relevant for group discrimination). All statistical treatment of data was carried out using the web-based MetaboAnalyst 5.0 software^29^.

### Ethics approval and patient consent statement

Written informed consent was obtained from all participants, and the study protocol was approved by the Research Ethics Board (approval ID number 10-0591) at the University Health Network (UHN; Toronto, ON, Canada) and conducted in accordance with the 1964 Declaration of Helsinki.

## Results

### Clinical and laboratory characteristics of patients

The clinical and pathological characteristics of the individuals in the sample are presented in Table 1. This study included a total of 40 subjects who were divided into four groups based on cancer type: 1) clear cell adenocarcinoma (CCC, *n* = 10); 2) endometrioid adenocarcinoma (EC, *n* = 10); 3) mucinous adenocarcinoma (MC, *n* = 9); and 4) serous cystadenocarcinoma (SeC, *n* = 10) and serous papillary cystic tumour of borderline malignancy (SeC, *n* = 1). Cancer staging was performed by board-certified pathologists at University Health Network (Toronto) who specialize in gynecologic oncology. Accordingly, patients were classified as being in FIGO stage 1 (*n* = 20), 2 (*n* = 5), 3 (*n* = 6), or 4 (*n* = 7), with 2 cases being unclassified. Finally, the participants’ CA125 levels were measured and dichotomized by their medians into four groups corresponding to the clinical stage of the disease.

### Serum metabolomic profiling

Extractions were performed on the collected serum samples using SPME extraction phases designed for either hydrophilic or hydrophobic analytes. Following extraction, the SPME extracts were analyzed on the UHPLC-HRAM (high-resolution, accurate-mass) platform in both positive and negative ion modes. Such comprehensive profiling enabled a broad range of metabolites/lipids to be captured, while excluding components such as proteins (macromolecules) not intended for analysis. QC samples and internal standards were used to verify technical precision and repeatability within analytical batches. The QC samples were clustered together in the relevant PLS-DA score plots (Supplementary Figs. 1, 3, and 5), and more than 90% of the coefficients of variation (CVs) for the selected features were below 30%, indicating good instrument reproducibility during the entire batch analysis and high quality of data, regardless of the applied chromatographic mode.

**Figure 1:**
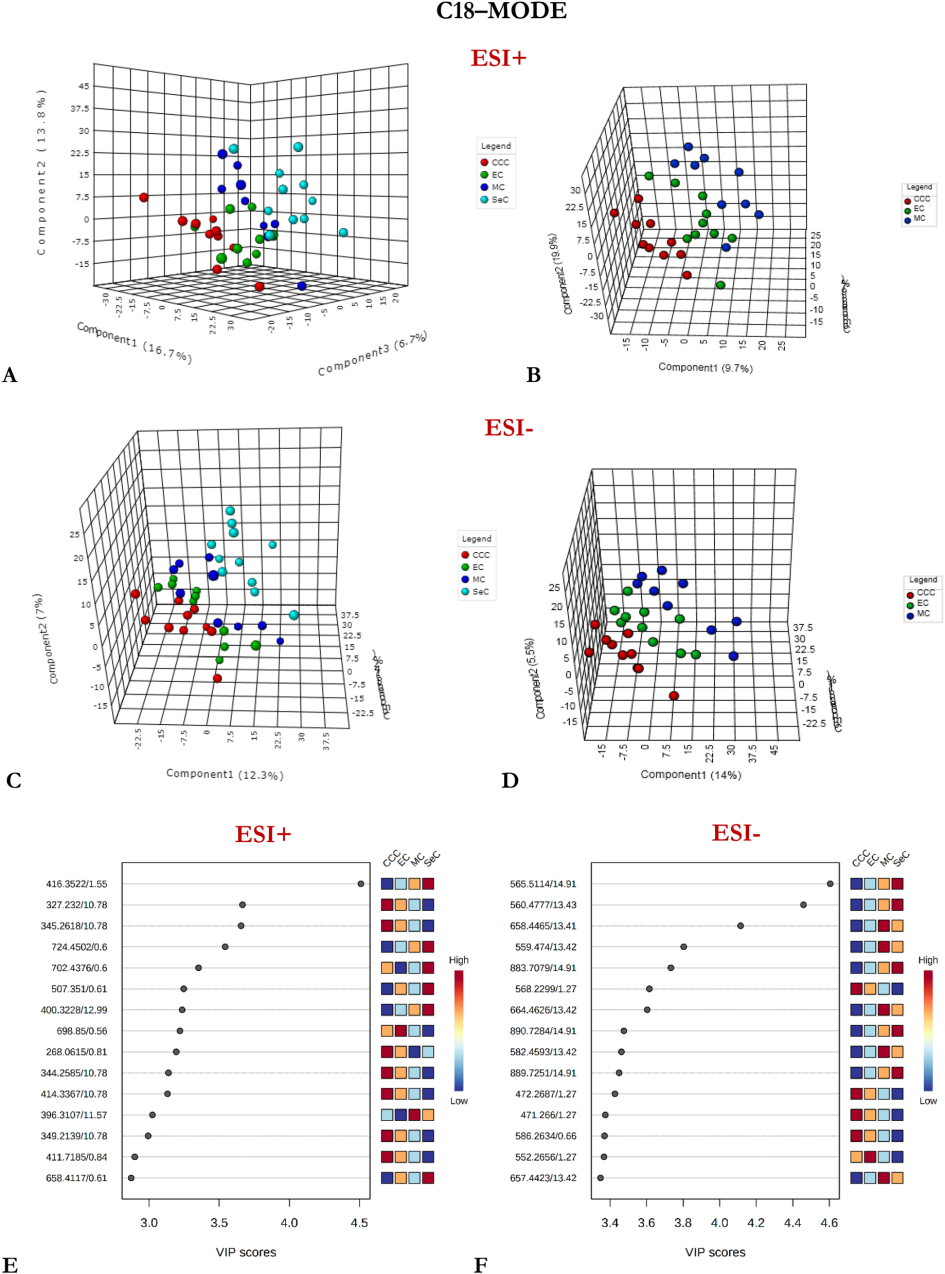
Three-dimensional PLS-DA score plots for features detected in positive (**A**, **B**) and negative (**C**, **D**) ion/reversed-phase (**C18-based phase**) mode and their corresponding VIP values (**E**, **F**). Red: clear cell carcinoma patients. Green: endometrioid carcinoma patients. Dark blue: mucinous carcinoma patients. Light blue: ovarian serous carcinoma patients. The following data-filtering parameters were used during analysis: RSD of QC samples < 30%; average of pooled QC samples over blanks ratios > 5; and total number of features (ions with unique m/z (mass-to-charge ratio) and retention-time values) = 1564 (ESI +) and 1798 (ESI-). Clear discrimination between lipidomic patterns in samples collected from patients with serous and non-serous carcinomas was observed. VIP schematic scores of PLS-DA analyses for clear cell (CCC) vs endometrioid (EC) vs mucinous (MC) vs serous (SeC) carcinoma (**E**, **F**). The labels: component 1, 2 and 3 along the axes represent the scores (the first three latent variables) of the model, which are sufficient to build a satisfactory classification model. Latent variables were calculated as a linear combination of the associated manifest variables. The example variables/metabolic features located within a first component and contributing the most to separation between study groups were presented in relevant VIP score plots. ESI + , positive ion mode; ESI-, negative ion mode.

**Figure 2:**
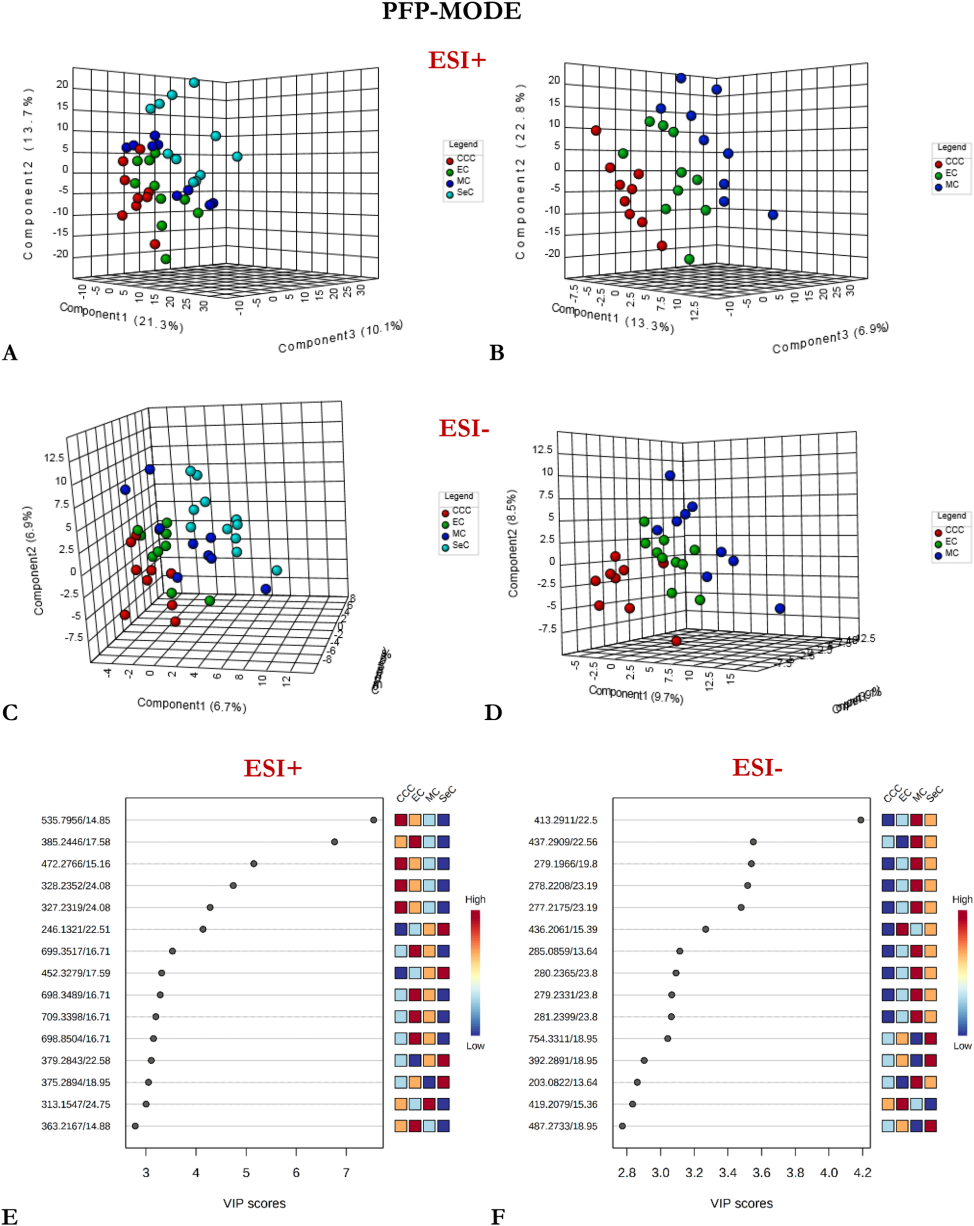
Three-dimensional PLS-DA score plots for features detected in positive (**A**, **B**) and negative (**C**, **D**) ion/reversed-phase (**PFP-based phase**) mode and their corresponding VIP values (**E**, **F**). Red: clear cell carcinoma patients. Green: endometrioid carcinoma patients. Dark blue: mucinous carcinoma patients. Light blue: ovarian serous carcinoma patients. The following data-filtering parameters were used during analysis: RSD of QC samples < 30%; average of pooled QC samples over blanks ratios > 5; and total number of features left for the analysis = 1047 (ESI+) and 458 (ESI-). Similar to the data obtained using the previous acquisition mode (reversed-phase/C18-based), a clear discrimination can be observed between metabolomic patterns for samples collected from serous and non-serous ovarian cancer patients. VIP schematic scores of PLS-DA analyses for clear cell (CCC) vs endometrioid (EC) vs mucinous (MC) and vs serous (SeC) carcinoma (**E**, **F**). The labels: component 1, 2 and 3 along the axes (the first three latent variables) represent the scores of the model, which are sufficient to build a satisfactory classification model. The example variables/metabolic features located within a first component and contributing the most to separation between study groups were presented in relevant VIP score plots. ESI+, positive ion mode; ESI-, negative ion mode.

**Figure 3:**
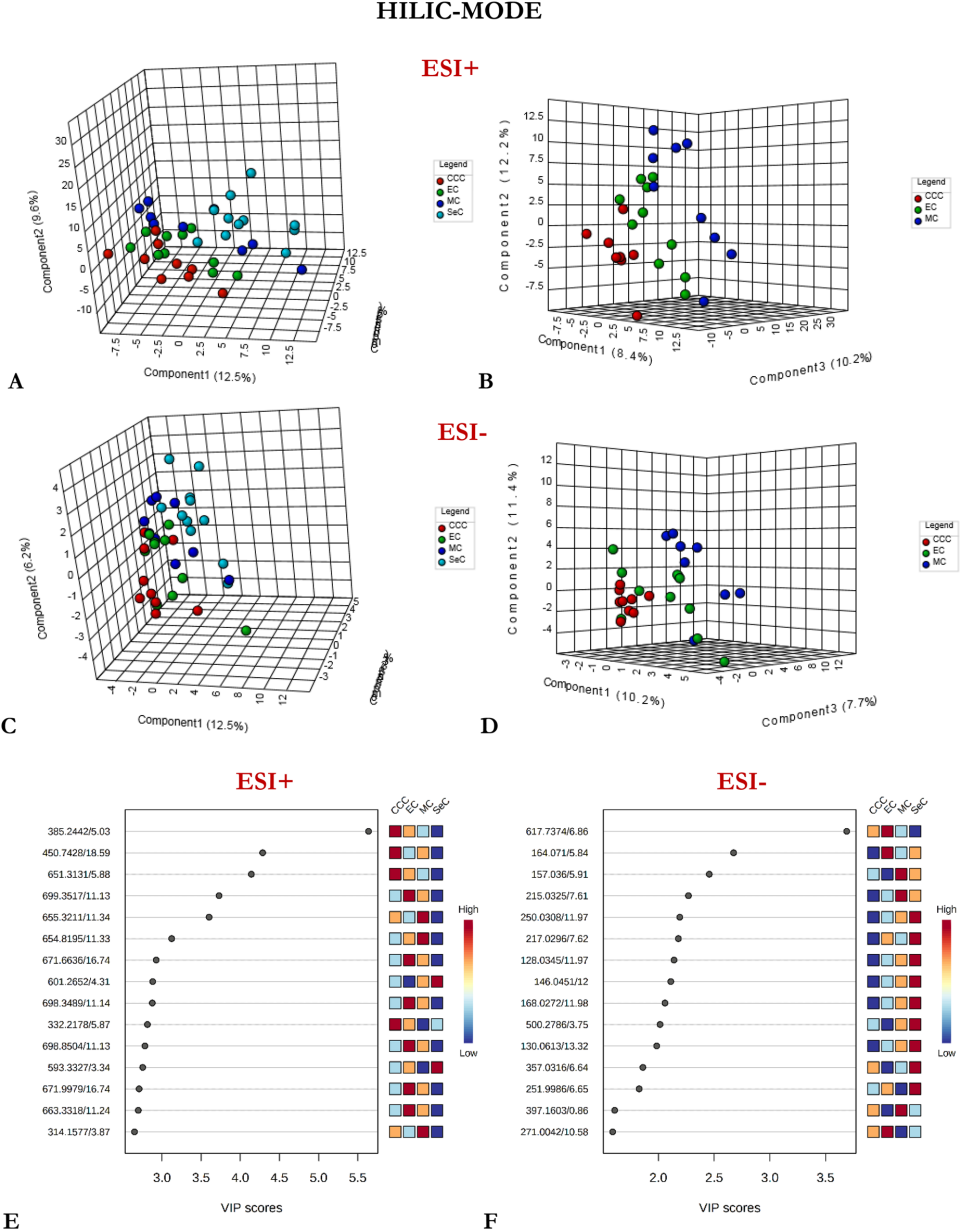
Three-dimensional PLS-DA score plots for features detected in positive (**A**, **B**) and negative (**C**, **D**) ion/**HILIC** mode and their corresponding VIP values (**E**, **F**). Red: patients affected by clear cell carcinoma. Green: endometrioid carcinoma patients. Dark blue: mucinous carcinoma patients. Light blue: ovarian serous carcinoma patients. The following data-filtering parameters were used during analysis: RSD of QC samples < 30%; average of pooled QC samples over blanks ratios > 5; total number of features for analysis = 540 (ESI+) and 147 (ESI-). For data collected with HILIC mode, the differentiation in the metabolomic patterns of serous and non-serous OC samples may be observable. VIP schematic scores of PLS-DA analyses for clear cell (CCC) vs endometrioid (EC) vs mucinous (MC) and vs serous (SeC) carcinoma (**E**, **F**). The labels: component 1, 2 and 3 along the axes (the first three latent variables) represent the scores of the model, which are sufficient to build a satisfactory classification model. The example variables/metabolic features located within a first component and contributing the most to separation between study groups were presented in relevant VIP score plots. ESI+, positive ion mode; ESI-, negative ion mode.

Supervised PLS-DA analysis was then performed on the entire dataset to assess variations in the measured metabolomes based on phenotype. Several PLS-DA models with different acquisition modes and coating types were constructed using either a four-class or two-class input: (1) CCC vs. EC vs. MC vs. SeC (Figs. 1, 2, 3), or (2) non-serous vs. serous carcinoma (NSeC vs. SeC) (Supplementary Figs. 2, 4, and 6). The resultant PLS-DA score plots showed a clear and significant difference between the metabolite profiles of the SeC patients and those of the NSeC cases (Figs. 1, 2, 3, Supplementary Figs. 2, 4, and 6). Similarly, a distinct trend toward separation among NSeC subgroups can be seen on the 3D score plots—with differences in lipidomic pattern being the most pronounced—likely as a result of SPME's ability to capture a greater diversity of lipid classes/subclasses compared to traditional extraction methods^30^ (Figs. 1, 2, 3B, D). Indeed, the minimal matrix effects associated with SPME enabled the detection of a large number of low-abundance species, such as phosphatidic acids, phosphatidylglycerols, (hexosyl)ceramides, lysophospholipids, which may be of particular importance in studies aimed at low-level biomarker discovery. To avoid overfitting the generated PLS-DA models, and to evaluate their predictive capability, a cross-validation procedure and testing with 100 random permutations were employed. The results of both tests confirmed that the proposed EOC subtype classification approach is statistically valid and provides significant predictive power.

### Selection of significant features which classify EOC subtypes

To identify the features responsible for the measured variance in the PLS-DA prediction models, VIP scores and FDR-adjusted P values were calculated and screened using significance thresholds of VIP ≥ 1.5 and/or *P* < 0.05. These thresholds were satisfied by 27 variables in C18-based chromatographic mode when C18-SPME microprobes were applied; 23 variables in PFP-based mode when MM-SPME microprobes were used; and 15 variables in HILIC mode when MM-SPME probes were used (Table 2). Notably, some discriminative metabolic features were detected in both ion modes (e.g., 18-hydroxycorticosterone, aldosterone, tryptophan, and glycocholic acid (PFP-based mode)), or in different acquisition modes (e.g., 4,5-dehydrodocosahexaenoic acid, monoacylglycerol (20:4), aldosterone, tryptophan, and androsterone sulfate). Various lipid mediators, mono-, di-, and triacylglycerols, ceramides, fatty acyl lipid classes, acylcarnitines, tryptophan metabolites, diverse bile acids classes, and intermediates in aldosterone synthesis were among those with the greatest effect on EOC subtype differentiation.

**Table 2:** Levels of metabolites or lipid species found to be most discriminative for ovarian carcinoma subtype classification.

Name^a^	m/z	Rt (min)	Adduct	Mode	VIP	FC CCC/EC	FC CCC/MC	FC EC/MC	FC NSeC/SeC
4,5-Dehydrodocosahexaenoic acid	327.2320	10.78	[M+H]^+^	C18/ESI+	3.67	1.49	2.46	1.65	1.95
	344.2585	10.78	[M+NH_4_]^+^	C18/ESI+	3.14	1.44	2.39	1.66	1.94
Monoacylglycerol (20:4)	396.3107	11.57	[M+NH_4_]^+^	C18/ESI+	3.02	1.21	0.32	0.27	0.59
Tetradecanoylcarnitine	372.3108	11.55	[M+H]^+^	C18/ESI+	2.87	0.62	0.29	0.46	0.56
3,5-Tetradecadiencarnitine	368.2796	3.11	[M+H]^+^	C18/ESI+	2.37	1.03	0.75	0.73	0.71
Monoacylglycerol (18:2)	377.2665	11.54	[M+Na]^+^	C18/ESI+	2.36	0.63	0.26	0.41	0.44
9-Hexadecenoylcarnitine	398.3265	7.67	[M+H]^+^	C18/ESI+	2.20	0.74	0.72	0.97	0.64
Lysophosphatidylethanolamine (22:6)	526.2929	9.58	[M+H]^+^	C18/ESI+	2.00	0.95	0.80	0.84	0.62
TG (triacylglycerol) (60:4)	984.8958	31.47	[M+NH_4_]^+^	C18/ESI+	1.89	0.35	0.75	2.17	0.80
TG (triacylglycerol) (59:6)	966.8488	30.22	[M+NH_4_]^+^	C18/ESI+	1.83	0.48	0.60	1.23	0.98
17-keto-docosahexaenoic acid	360.2534	6.41	[M+NH_4_]^+^	C18/ESI+	1.68	1.47	1.50	1.02	1.01
TG (triacylglycerol) (54:5)	903.7413	30.83	[M+Na]^+^	C18/ESI+	1.67	0.42	0.68	1.64	0.83
DG (diacylglycerol) (36:3)	601.5191	31.26	[M+H−H_2_O]^+^	C18/ESI+	1.58	0.54	0.65	1.19	0.91
HexCer (hexosylceramide) (d49:1)	932.7916	27.97	[M+Na]^+^	C18/ESI+	1.57	0.40	0.75	1.88	0.80
TG (triacylglycerol) (52:3)	874.7856	31.26	[M+NH_4_]^+^	C18/ESI+	1.54	0.60	0.79	1.30	0.92
TG (triacylglycerol) (54:3)	907.7727	32.17	[M+Na]^+^	C18/ESI+	1.51	0.58	0.80	1.39	0.85
Linoleic acid	559.474	13.42	[2M−H]^−^	C18/ESI−	3.80	0.45	0.29	0.66	0.39
	581.4558	13.42	[2M−2H+Na]^−^	C18/ESI−	3.07	0.58	0.39	0.66	0.59
TG (triacylglycerol) (55:7)	889.7251	14.91	[M−H]^−^	C18/ESI−	3.45	0.66	0.46	0.69	0.64
Cardiolipin (CL) (76:14)	745.4627	13.42	[M−2H]^2−^	C18/ESI−	3.28	0.64	0.43	0.66	0.64
*N*-arachidonoyl taurine (C20:4-NAT)	470.2623	1.27	[M+CH_3_COO]^−^	C18/ESI−	3.27	0.99	1.18	1.19	2.29
Phosphatidic acid (PA) (P-38:6)	739.4458	13.42	[M+Cl]^−^/[2M+2Cl]^2−^	C18/ESI−	3.10	0.60	0.41	0.68	0.65
Ceramide-phosphoethanolamine (PE-Cer) (d32:1)	965.7111	14.91	[3M−H+Cl]^2−^	C18/ESI−	3.09	0.66	0.47	0.70	0.68
9-OAHSA/12-OAHSA/9/12-(Oleoyloxy)stearic acid	563.5047	14.91	[M−H]^−^	C18/ESI−	3.00	0.46	0.45	0.97	0.59
PG (phosphatidylglycerol) (P-29:0)	663.4591	13.42	[M−H]^−^	C18/ESI−	2.99	0.60	0.40	0.67	0.58
Stearidonic acid/6,9,12,15-Octadecatetraenoic acid	275.2017	10.83	[M−H]^−^	C18/ESI−	2.74	0.97	0.65	0.67	0.35
TG (triacylglycerol) (60:12)	971.7281	14.91	[M+Cl]^−^/[2M+2Cl]^2−^	C18/ESI−	2.60	0.82	0.55	0.67	0.76
15-hydroperoxy-11,13-eicosadienoic acid (15-HpEDE)	339.2544	13.42	[M−H]^−^	C18/ESI−	2.28	0.72	0.52	0.71	0.62
Glycochenodeoxycholate-3/7-sulfate	263.6282	0.57	[M−2H]^2−^	C18/ESI−	1.76	4.90	2.32	0.47	0.94
Sphingosine-1-phosphate (t16:1)	385.2446	17.58	[M+NH_4_]^+^/[2M+H+Na]^2+^	PFP/ESI+	6.77	0.85	1.12	1.32	3.45
4,5-Dehydrodocosahexaenoic acid	327.2319	24.08	[M+H]^+^	PFP/ESI+	4.28	1.58	2.62	1.66	1.99
Dihydroxypregn-4-en-3-one 20-glucosyl-(1-4)-6-acetyl-glucoside	699.3517	16.71	[M+H]^+^	PFP/ESI+	3.53	0.74	0.95	1.30	2.66
Monoacylglycerol (20:4)	379.2843	22.58	[M+H]+	PFP/ESI+	3.10	0.86	0.37	0.43	0.59
3α/β-Hydroxy-5-cholenoic acid	375.2894	18.95	[M+H]^+^	PFP/ESI+	3.05	1.35	1.20	0.89	0.47
LPI (Lysophosphatidylinositol) (O-32:1)	313.1547	24.75	[M+H+K]^2+^/[M+2ACN+2H]^2+^	PFP/ESI+	3.00	0.92	0.79	0.86	1.64
18-Hydroxycorticosterone	363.2167	14.88	M+H]^+^	PFP/ESI+	2.78	0.65	1.02	1.56	2.68
	421.2234	14.91	[M+CH_3_COO]^−^	PFP/ESI−	2.72	0.77	1.10	1.43	2.43
Aldosterone	361.201	15.34	[M+H]^+^	PFP/ESI+	2.47	0.84	0.99	1.18	1.76
	419.2079	15.36	[M+CH_3_COO]^−^	PFP/ESI−	2.83	0.88	1.04	1.19	1.93
5α/β-Choladien-24-oic acid	357.2789	18.96	[M+H]^+^	PFP/ESI+	2.73	1.09	1.10	1.01	0.48
l-Kynurenine	209.0922	11.97	[M+H]^+^	PFP/ESI+	2.58	0.59	0.64	1.08	0.69
F4-Neuroprostane (4-series)	379.2488	12.68	[M+H]^+^	PFP/ESI+	2.00	1.62	1.07	0.66	0.16
Chenodeoxycholic acid glycine conjugate	450.3214	17.58	[M+H]^+^	PFP/ESI+	1.86	1.57	1.27	0.81	0.36
	472.3033	17.58	[M+Na]^+^	PFP/ESI+	1.76	1.81	1.34	0.74	0.39
3,7-Dihydroxy-6/12-oxo-5α/β-cholan-24-oic acid	448.3057	16.02	[M+ACN+H]^+^	PFP/ESI+	1.67	2.37	0.69	0.29	0.50
	448.3057	16.93	[M+ACN+H]^+^	PFP/ESI+	1.55	3.41	2.15	0.63	0.46
3-Hydroxycapric acid	189.1487	15.46	[M+H]^+^	PFP/ESI+	1.63	1.71	3.17	1.85	0.43
Monoacylglycerol (18:2)	413.2911	22.50	[M+CH_3_COO]^−^	PFP/ESI−	4.19	0.80	0.37	0.46	0.59
Octadecatrienoic acid, FA (18:3)	277.2175	23.19	[M−H]^−^	PFP/ESI−	3.48	1.10	0.41	0.37	0.67
Acetyl-*N*-formyl-5-methoxykynurenamine (AFMK)	285.0859	13.64	[M+Na−2H]^−^	PFP/ESI−	3.11	0.65	0.41	0.64	0.72
Octadecadienoic acid, FA (18:2)	279.2331	23.8	[M−H]^−^	PFP/ESI−	3.07	1.13	0.59	0.52	0.68
l-Tryptophan	203.0822	13.64	[M−H]^−^	PFP/ESI−	2.86	0.65	0.45	0.68	0.70
	205.0974	14.42	M+H]^+^	PFP/ESI+	2.47	0.61	0.49	0.80	0.79
Dihydroxycholanoic acid	391.2857	18.95	[M−H]^−^	PFP/ESI−	2.08	1.21	1.09	0.90	0.57
*N*-acyltaurine, NAT (19:0)	464.3019	15.79	[M+CH_3_COO]^−^	PFP/ESI−	1.78	2.16	0.67	0.31	0.52
Androsterone sulfate	369.1743	14.92	[M−H]^−^	PFP/ESI−	1.54	0.51	0.52	1.01	2.07
Glycocholic acid	446.2916	16.73	[M−H_2_O−H]^−^	PFP/ESI−	1.51	2.81	1.83	0.65	0.45
	466.3163	16.02	[M+H]^+^	PFP/ESI+	1.53	2.16	0.68	0.32	0.51
Phosphatidic acid (PA) (38:4)	385.2442	5.03	[M+2Na]^2+^	HILIC/ESI+	5.64	0.90	1.13	1.26	3.92
Phosphatidylserine (PS) (42:10)	450.7428	18.59	[M+2Na]^2+^	HILIC/ESI+	4.29	2.58	2.02	0.78	3.70
S-Adenosylmethionine	416.1718	2.64	[M+NH_4_]^+^	HILIC/ESI+	2.34	0.96	0.93	0.97	0.033
l-Tryptophan	205.0972	8.14	[M+H]^+^	HILIC/ESI+	2.12	0.65	0.49	0.75	0.85
Xanthine	153.0406	2.26	[M+H]^+^	HILIC/ESI+	2.04	0.80	0.64	0.80	0.86
Indolelactic acid	188.0706	8.14	[M+H−H_2_O]^+^	HILIC/ESI+	1.99	0.72	0.49	0.67	0.84
5-Hydroxytryptophan	133.0317	8.09	[M+2Na]^2+^	HILIC/ESI+	1.47	1.35	1.04	0.77	1.32
l-Phenylalanine	164.071	5.84	[M−H]^−^	HILIC/ESI−	2.67	0.76	0.78	1.03	0.86
Allantoic acid	157.036	5.91	[M−H_2_O−H]^−^	HILIC/ESI−	2.46	1.25	0.47	0.38	1.38
l-Glutamate	128.0345	11.97	[M−H_2_O−H]^−^	HILIC/ESI−	2.14	0.73	0.75	1.03	0.89
	146.0451	12	[M−H]^−^	HILIC/ESI−	2.11	0.76	0.79	1.04	0.89
	168.0272	11.98	[M+Na−2H]^−^	HILIC/ESI−	2.06	0.77	0.80	1.03	0.86
Lysophosphatidylethanolamine (LPE) (20:4)	500.2786	3.75	[M−H]^−^	HILIC/ESI−	2.01	1.26	0.77	0.61	0.82
Creatine	130.0613	13.32	[M−H]^−^	HILIC/ESI−	1.98	0.84	0.71	0.84	0.95
Aldosterone	395.1632	0.86	[M+Cl]^−^	HILIC/ESI−	1.61	1.09	0.97	0.89	1.33
l-Glutamine	127.0504	12.93	[M−H_2_O−H]^−^	HILIC/ESI−	1.59	1.31	1.11	0.84	1.01
Androsterone sulfate	369.1741	0.58	[M−H]^−^	HILIC/ESI−	1.46	0.56	0.50	0.91	2.01

Metabolites/lipids were selected according to VIP values (≥ 1.5) and/or Mann–Whitney test (adjusted *p* < 0.05) results. Fold changes (FCs) were the ratios of the average MS ion intensities (peak areas) between particular groups studied.

CCC – clear cell carcinoma, EC – endometrioid carcinoma, MC – mucinous carcinoma, NSeC, SeC – non-serous and serous ovarian carcinoma, respectively.

^a^Metabolites or lipids were sorted out with decreasing VIP values and data acquisition mode.

**Figure 4:**
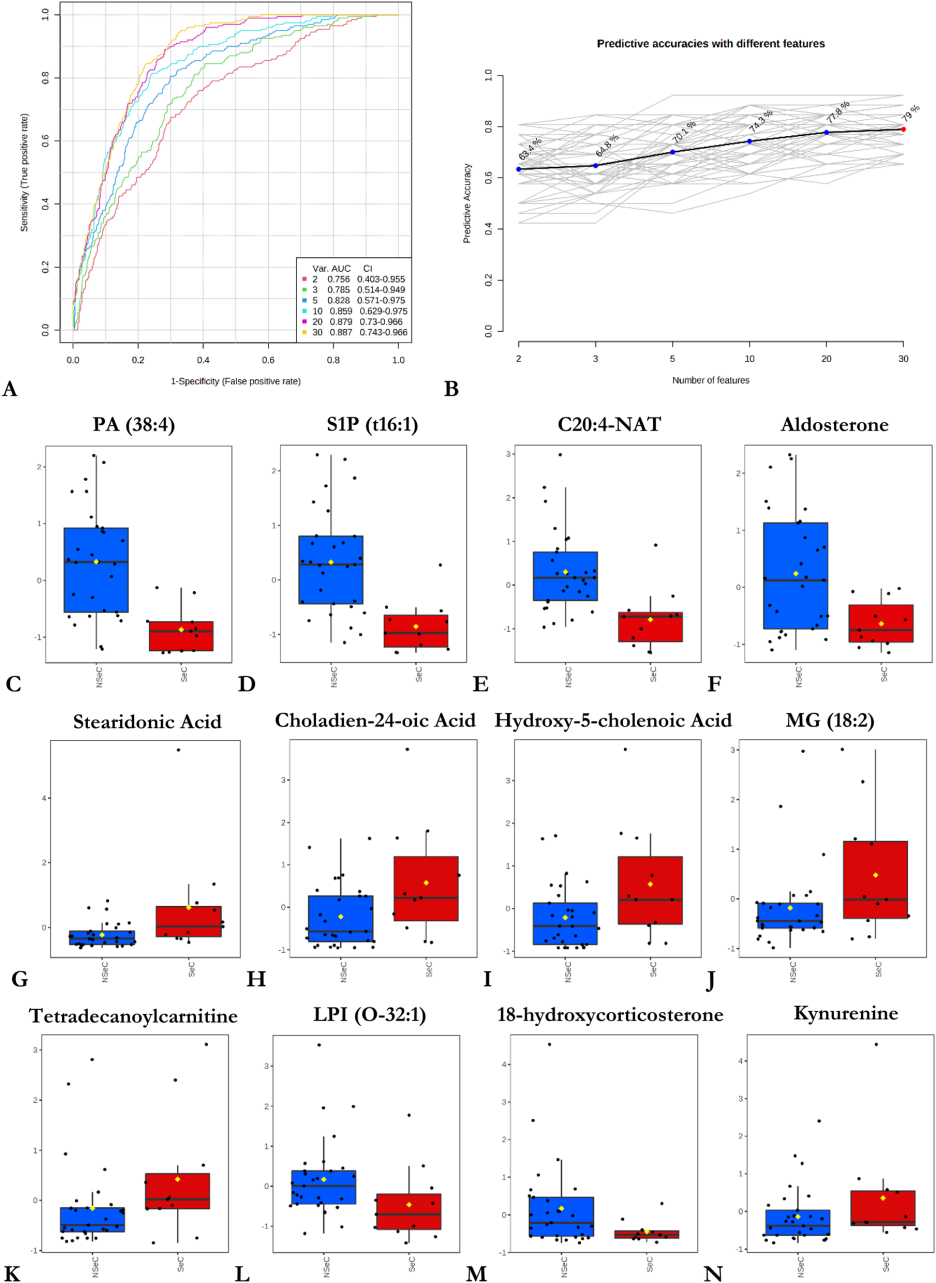
ROC curve analysis based on a multivariate PLS-DA algorithm for *n* = 30 independent metabolite features discriminating serous and non-serous carcinomas with VIP ≥ 2.0, and predictive accuracy model with a different number of features involved (**A**, **B**). Box whisker plots of the twelve most significant metabolites or lipids in the analysis of variance results for NSeC, non-serous carcinoma (NSeC, blue boxes) and serous carcinoma (SeC, red boxes). The x-axis depicts the specific metabolite/OC group, and the y-axis presents the normalized peak intensity (**C**–**N**). The lower, middle, and upper lines in the relevant box plots correspond to the 25th, 50th (the median), and 75th percentiles.

Next, receiver operating characteristic (ROC) analysis was performed on variables with a VIP score ≥ 2 to evaluate their predictive ability in the discrimination of serous and non-serous OC subtypes. 30 analytes out of 57 putatively annotated compounds (Table 2) met this threshold and were involved in the analysis. Since the ROC analysis of individual metabolic features is unable to assess the relationships that account for the observed variance between them, a multivariate ROC analysis approach was implemented based on the PLS-DA method. Figure 4A and B shows the ROC curves and the predictive accuracy model for the examined features. We found that the model containing 20 features showed excellent predictive performance, with an ROC AUC (area under the curve) value of > 88%. In addition, 12 significant features were selected based on their average importance in group classification and displayed in relevant box plots in Fig. 4C–N. As can be seen, the greatest changes can be observed in cellular lipid mediators involving phosphatidic acid (38:4) and lysophosphatidylinositol (O-32:1), tryptophan catabolites, bile acids, and components of aldosterone synthesis pathways when serous OC metabolic phenotypes were compared against non-serous metabolic phenotypes. Additionally, to verify how levels of 30 above-mentioned distinguishing metabolites/lipids are changing as the disease progresses, ROC curves for discriminating early-stage patients (e.g. stage I and II) and advanced-stage OC patients (stage III and IV) were constructed with results presented in Supplementary Fig. 7. Classifiers distinguishing early-stage from advanced disease stage had an AUROC of 0.8 thus confirming the accuracy of classification of the study groups/samples when multivariate ROC curve analysis was yet employed on the set of 20 analytes.

## Discussion

At present, strategies for the early diagnosis and personalized treatment of EOC are limited^3,4^. As such, the development of non-invasive methods that enable the precise classification of EOC subtypes is critical for cancer management, as such approaches would provide accurate information about clinical tumor behavior, its prognosis, and the most effective treatments for the disease^31^. Although diagnostic accuracy relating to several histologically distinct types of OC tumor has improved significantly over the last decade, biopsy is still often the only option, as pre-diagnostic testing continues to suffer from a lack of sensitivity and specificity^32^. To remedy this limitation, it will be highly important to explore new biomarkers in combination with statistical learning algorithms that are able to consider additional risk factors. Fortunately, this goal has become feasible due to recent technological progress with respect to sample-pretreatment techniques for the extraction of a very wide range of stable and unstable analytes, as well as advancements in the speed and resolution of LC–MS metabolomic analysis.

In this research, we demonstrate the feasibility of our SPME-HRAM technique for classifying the four main EOC subtypes via comprehensive metabolite/lipid species profiling and the identification of the metabolomic signature ascribed to a given histopathologic phenotype. To capture and analyze the entire metabolome at once, we tested two types of SPME coatings (i.e., C8/benzenesulfonic acid (C8/SCX) and C18) along with three acquisition modes. This design enabled the identification of over 1000 metabolites/lipid species for each EOC subtype. Our data imply that metabolic reprogramming mechanisms are different between EOC subtypes, as significant alterations were noted in the following pathways: energy metabolism; tryptophan catabolism; bile acid metabolism; lipid (glycerophospholipid, lysophospholipid, and neutral) metabolism; steroid hormone (androgen and aldosterone) metabolism; and polyunsaturated fatty acid (docosahexaenoic acid, linoleic acid, linolenic acid, and stearidonic acid) metabolism.

Specifically, our data demonstrate distinct differences in the energy metabolism patterns of the EOC subtypes, mainly in relation to glutamine (Gln) and fatty acid (FA) metabolism. It is widely accepted that tumor hypoxia and cancer-associated mutations affect central carbon metabolism and enhance cancer cells’ dependence on glutamine for their growth/division^33^. CCC is a unique EOC histotype in this regard, as it is more dependent on aerobic glycolysis than other subtypes. This feature allows CCC to be more resistant to chemotherapy, which results in higher cell survival under conditions wherein mitochondrial function is suppressed, thus diminishing ROS generation^34^. Our data appear to support this observation, as the highest amounts of Gln were observed in the CCC samples, along with significantly reduced glutamate content. Adding to the above, the CCC samples showed increased levels of N-acyl taurines (in particular, nonadecanoyl-taurine), which function as endogenous lipid messengers that improve glucose homeostasis^35^, further pointing to the glucose-dependent phenotype of these cells. In contrast, the accumulation of several long-chain acylcarnitines (i.e., tetradecanoylcarnitine, 3,5-tetradecadiencarnitine, and 9-hexadecenoylcarnitine) was observed in the SeC samples, which may suggest increased FA metabolism in this histotype. Even though the heavy dependence of cancer cells on glutamine and glucose is well-recognized, it is common for such cells to rewire their metabolism in order to sustain the production of ATP and the macromolecules required for cell growth, proliferation, and survival^33,36^.

Another interesting alteration of the EOC metabolome was accelerated tryptophan (Trp) catabolism. Although enhanced Trp breakdown and elevated kynurenine (Kyn) concentrations is a common indicator of tumor progression—and, ultimately, poor clinical outcomes—in cancer patients^37,38^, no prior research has examined the distinct differences in the Kyn/Trp ratios of the EOC subtypes, or the significance of alternative paths of Trp degradation in indolelactic acid and acetyl-N-formyl-5-methoxykynurenamine (AFMK) accumulation and how they contribute to clinical outcomes. Researchers have explored a variety of tumor immune escape mechanisms^39^, and clinical studies have proven that the immunoregulatory pathway of Trp catabolism that modulates the immunosuppressive microenvironment constitutes a fundamental trait of cancer progression^38,39^. Several strategies targeting Trp catabolism and the production of immune suppressive catabolites have been tested in clinical trials, but the results of these studies have been inconclusive^38,40^. A better understanding of the changes in Trp catabolites and how they contribute to cancer progression may prove beneficial to EOC patients, as it could enable more personalized treatment regimens, thereby significantly improving quality of life.

Apart from alterations in amino acid and energetic fuel metabolism, our findings also revealed apparent differences in the levels of numerous bile acids, which supports previous findings relating to their critical role in carcinogenesis^41^. Although several studies have demonstrated decreased bile acid biosynthesis in EOC patients, none have explored how this process differs between EOC histologic phenotypes and whether these differences can serve as an accurate metric for discriminating between them^42–44^. Intriguingly, the serum samples from the CCC patients were characterized by much higher concentrations of bile acids compared to the other histotypes, again suggesting that this form of OC possesses heightened resistance to challenging microenvironments containing higher amounts of pro-apoptotic agents^41^.

The deregulation of lipid metabolism (beyond energy fuel utilization) constitutes another critical factor in OC^45,46^. Indeed, numerous dysregulated lipid species have been detected in EOC samples, suggesting that distinct differences in the pattern of these alterations may function as a signature of a given EOC histotype. The integral role of FAs in tumorigenesis has been well established, particularly their association with cancer cells’ increased reliance on de novo FA biosynthesis and exogenous FA uptake in order to satisfy the metabolic requirements of cell proliferation and to provide essential energetic fuel under conditions of metabolic stress^36^. Furthermore, researchers studying EOC patients have observed distinct changes in the fluidity of biological membranes and the patterns of lipid mediators—either functioning as secondary messengers in various intracellular-signaling pathways, or engaging in the remodeling of the entire tumor microenvironment via paracrine-signaling mechanisms—which further points to the essential role of the lipid pool’s vast structural diversity in carcinogenesis^46,47^. Thus, when targeting FA metabolism in cancer treatment, is necessary to consider the complex framework within which FAs and their intermediates/by-products are synthesized and exert their functions, including several compensatory or interconnected pathways activated to sustain FA metabolism, as well as the dynamic interactions within the cancer microenvironment.

Finally, accelerated steroid hormone (i.e., androsterone and aldosterone) biosynthesis has been found in non-serous EOC subtypes. Though the role of steroids in ovarian cancer has yet to be clearly defined, several studies have demonstrated that they can be produced by malignant ovarian tumors in addition to the adrenal glands^48^. Adding to the above, high plasma aldosterone levels have been observed in the advanced stages of OC, likely due to primary aldosteronism^49^. Notably, the hyperaldosteronism regressed, and the patient’s hypertension improved following the surgical removal of the malignancy. Accordingly, a better understanding of alterations in steroid metabolism/signaling may play a key role in improving EOC diagnosis and treatment.

In conclusion, this paper presents an untargeted SPME-UHPLC/MS profiling pipeline intended to expand serum metabolome coverage and accurately classify EOC subtypes. Despite the significant differences in the levels of metabolites or lipid species between EOC subtypes, none of the individual metabolites or lipids independently allowed for correct and reliable classification given tumor heterogeneity that appears to be very high not only across subtypes but also within a particular single tumor type. However, integrating complex multi-analyte profiling and multivariate statistical analyses yielded an accurate and robust tool for identifying potential diagnostic biomarkers. Nonetheless, this study contains several limitations that should be addressed in future research. For instance, future studies could attempt to replicate our results using a larger sample, and validation studies are warranted to demonstrate whether the identified compounds have clinical utility in the diagnosis or management of EOC patients. Specifically, given the clinical significance of early diagnosis, as a next stage larger studies with independent cohorts of OC patients and control group (healthy subjects, benign disease) are planned to confirm the usefulness of the analytical protocol proposed to detect ovarian cancer at early stages in addition to being specific enough to distinguish particular histotypes thus corroborating our initial findings. This expanded study will not only increase the number of patients examined but will also involve detailed clinical characteristics of cases enrolled and investigations of potential impact of clinical variables on metabolomics findings identified to build strong conclusions on connection between affected metabolites/lipids and particular clinical phenotype. Finally, though our preliminary data provide a good direction towards future research further analytical improvements for a larger cohort follow-up study might be considered, such as implementation of robust tools for quality assurance (QA) and quality control (QC) in data collection, introducing additional technical replicates, effective data normalization methods to minimize batch effects, and applying unsupervised methods for proper data visualization, clustering and sample group discrimination. All these additional steps may provide a more supplemented and comprehensive approach for effective cancer screening and a better indication of discriminating metabolic biomarkers.

## Supplementary Information

Supplementary Information.

## Data Availability

The datasets supporting the findings of this study are available from the corresponding author upon reasonable request.
