# Supplementary material for: SPME-LC/MS-based serum metabolomic phenotyping for distinguishing ovarian cancer histologic subtypes: a pilot study
Source: Sci Rep. 2021 Nov 17;11:22428. doi: 10.1038/s41598-021-00802-9 (PMC8599860; doi:10.1038/s41598-021-00802-9)
Supplement: Supplementary file 1 — Supplementary Information. [file 41598_2021_802_MOESM1_ESM.docx]

**Supplementary Information**

**SPME-LC/MS-BASED SERUM METABOLOMIC PHENOTYPING FOR DISTINGUISHING OVARIAN CANCER HISTOLOGIC SUBTYPES: A PILOT STUDY**

Mariola Olkowicz^1#^, Hernando Rosales-Solano^1^, Vathany Kulasingam^2,3†^, Janusz Pawliszyn^1†^

^1^ Department of Chemistry, University of Waterloo, Waterloo, ON, N2L 3G1, Canada;

^2^ Department of Laboratory Medicine and Pathobiology, University of Toronto, Toronto, ON, M5S 1A8, Canada;

^3^ Division of Clinical Biochemistry, University Health Network, Toronto, ON, M5G 2C4, Canada.

† Corresponding authors:

vathany.kulasingam@uhn.ca (V. Kulasingam), Tel: +1 416-340-4800 8589, Fax: +1 416-340-4215;

janusz@uwaterloo.ca (J. Pawliszyn), Tel: +1 519-888-4641, Fax: +1 519-888-4348;

# current address: Jagiellonian Centre for Experimental Therapeutics (JCET), Jagiellonian University, Krakow, Poland.

**SUPPLEMENTARY METHODS**

**Supplementary Table 1.** LC method details as well as MS acquisition parameters for analysis of metabolites and lipid species in clinical serum samples.

| Ultra-High-Performance Liquid Chromatography | | | | |
| --- | --- | --- | --- | --- |
| Metabolomic Investigations Lipidomic Investigations  Reversed phase HILIC Reversed phase | | | | |
| Column | Supelco Discovery HS F5, 120 Å, 3 μm, 100 x 2.1 mm | | Merck SeQuant ZIC-HILIC, 200 Å, 3.5 μm, 100 x 2.1 mm | Waters XSelect CSH C18, 130Å, 3.5 µm, 75 mm x 2.1 mm |
| Mobile phase  Phase A      Phase B | H_2_O + 0.1% FA (+ 1 mM AA)  ACN + 0.1% FA (+ 1 mM AA) | | H_2_O + 0.1% FA (+ 1 mM AA)  ACN + 0.1% FA (+ 1 mM AA) | MeOH/H_2_O 40:60 + 10 mM AcNH_4_ + 1 mM AA (+ 0.02% AA)  IPOH/MeOH 90:10 +  10 mM AcNH_4_ + 1 mM AA (+ 0.02% AA) |
| Gradient programme | 0–3 min 0% B; 3–25 min 0–90% B; 25–34 min 90% B; 34–35 min 90–0% B; 35–40 min 0% B | | 0–2 min 90% B; 2–22 min 90–50% B; 22–27  min 50% B; 27–27.5 min 50–90% B; 27.5–35 min 90% B | 0–2 min 20% B; 2–4 min 20–30% B; 4–25 min 30–80% B; 25–35 min 80–85% B; 35–38 min 85–95% B; 38–41 min 95% B; 41-50 min 20% B |
| Flow rate [µL/min] | 300 | | 350 | 300 |
| Column temperature [°C] | 25 | | 40 | 55 |
| Autosampler temperature: [°C] | 4 | | 4 | 4 |
| Injection volume [µL] | 10 | | 10 | 10 |
| Thermo Exactive Mass Spectrometer (HESI ion source) | | | | |
| Acquisition mode Full Scan | | | | |
| Mass range [m/z] 100 – 1000 (for HILIC 75 – 1000) | | | | |
| Max. injection time [ms] 100 | | | | |
| Automatic gain control Balanced: 1e6 | | | | |
| Resolution High: 50,000 at 2 Hz (FWHM* 200 m/z) | | | | |
| Electrospray voltage, kV | | 4.0 (-2.9) | 3.5 (-3.2) | 3.5 (-2.9) |
| Sheath gas | | 55 | 60 | 30 |
| Auxiliary gas | | 30 | 20 | 10 |
| Sweep gas | | 5 | 2 | 2 |
| Capillary temperature [°C] | | 300 (300) | 275 (250) | 300 (300) |
| Vaporizer temperature [°C] | | 300 | 300 | 300 |
| Lock Mass m/z 391.2843 (ve+); m/z 255.2329 (ve-) | | | | |

FA – formic acid; AA – acetic acid; AcNH_4_ – ammonium acetate; ESI– mode parameters in brackets; *Full-width at half maximum (FWHM).

**A**
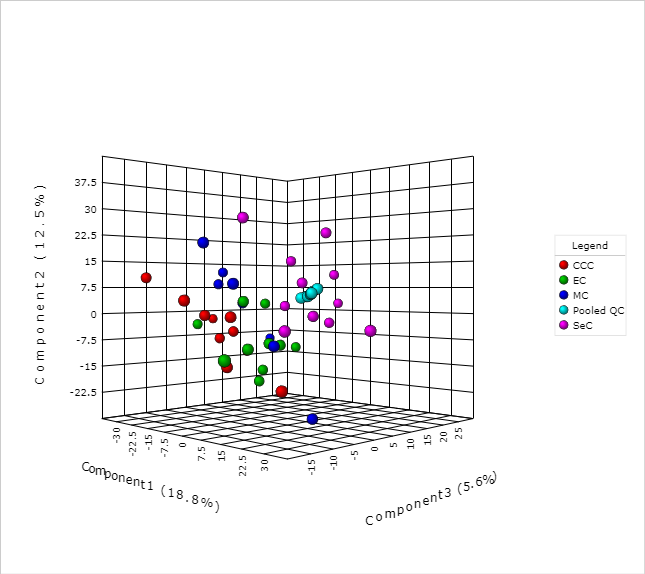
**B**
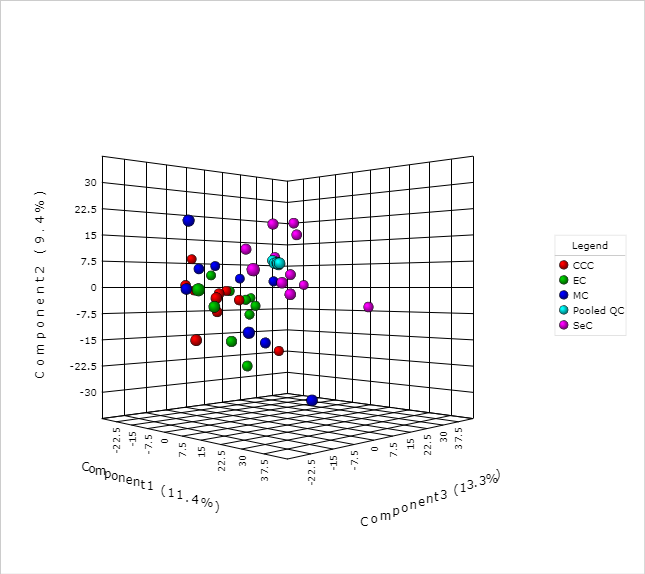


**ESI-**

**ESI+**

**Supplementary Fig. 1.** *Differences in serum lipidomic profiles in serous (SeC), endometrioid (EC), clear cell (CCC) and mucinous (MC) carcinoma samples observed in ESI+ and ESI- mode.* The 3D-PLS-DA plots show comparisons of the various groups. As can be seen, the pooled QC samples (injected every 8 sample runs) cluster very tightly, which strongly suggests instrument stability throughout the LC-MS runs. The degree of variance, which reflects the strength of the constructed model, is given in parentheses on each axis. The following data-filtering parameters were used for analysis: RSD of QC samples < 30%; average of pooled QC samples over blanks ratios > 5; and total number of features (ions with unique m/z (mass-to-charge ratio) and retention-time values) = 1564 (for ESI+ mode) and 1798 (for ESI- mode).

**A
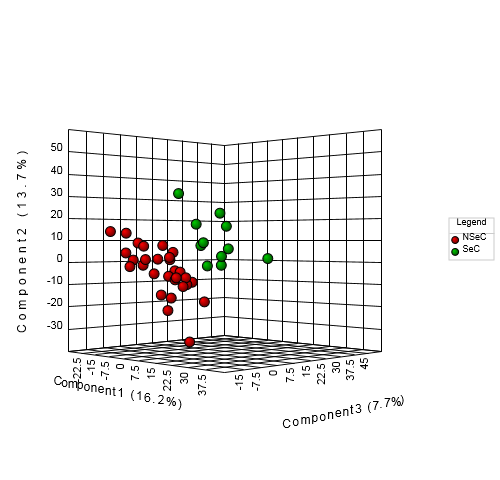
B
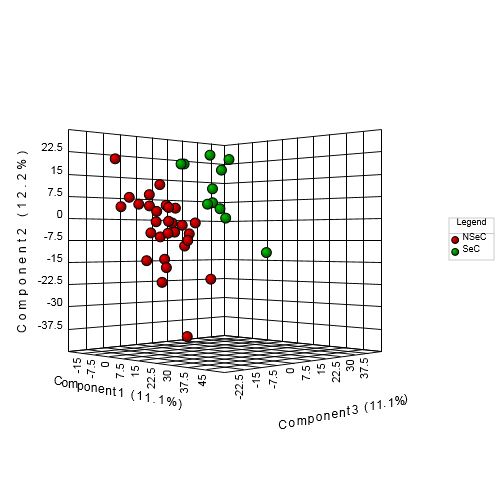
**

**ESI-**

**ESI+**

**Supplementary Fig. 2.** *Three-dimensional PLS-DA score plots for features detected in serous (SeC) and non-serous (NSeC) carcinoma samples (C18 reversed-phase mode).* **Green:** serous carcinoma samples. **Red:** non-serous carcinoma samples. A clear difference in the metabolomic patterns of these two histological subtypes can be observed.

**A**
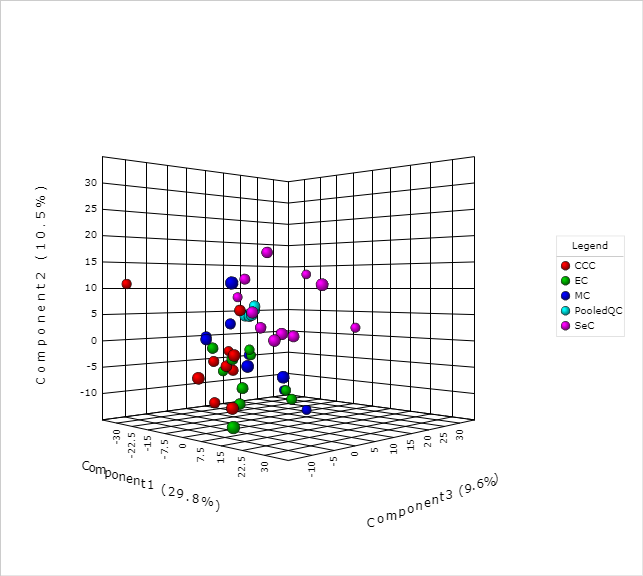
**B**
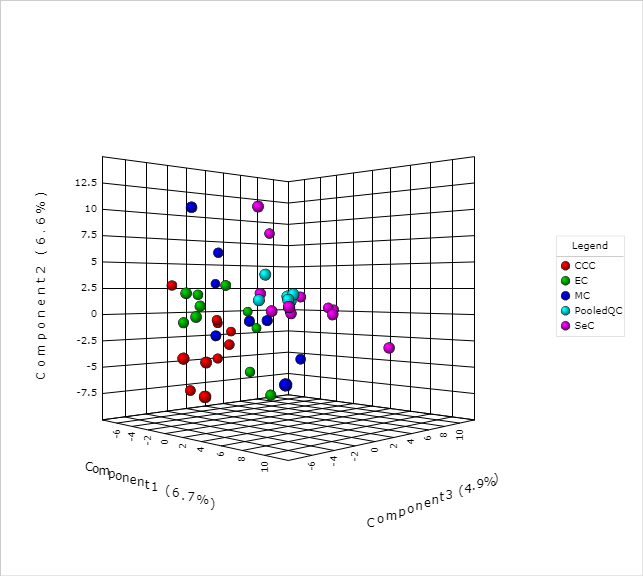


**ESI-**

**ESI+**

**Supplementary Fig. 3.** *Differences in serum metabolomic profiles in serous (SeC), endometrioid (EC), clear cell (CCC), and mucinous (MC) carcinoma samples observed in ESI+/PFP- and ESI-/PFP-based mode.* The 3D-PLS-DA plots show comparisons of the various groups. As can be seen, the pooled QC samples (injected every 8 sample runs) cluster close together demonstrating good instrument stability during the LC/MS run. The following data-filtering parameters were used for analysis: RSD of QC samples < 30%; average of each group over blanks ratios > 5; and total number of features = 1047 (for ESI+ mode) and 458 (for ESI- mode).

**A**
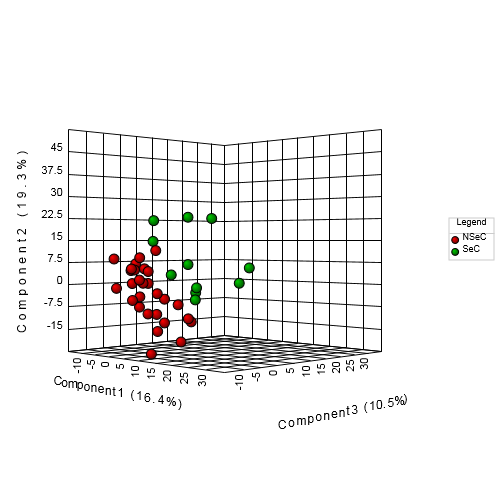
**B
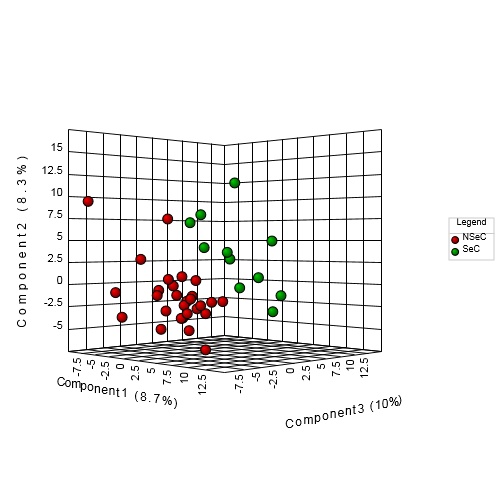
**

**ESI-**

**ESI+**

**Supplementary Fig. 4.** *Three-dimensional PLS-DA score plots for features detected in serous (SeC) and non-serous (NSeC) carcinoma samples (PFP-based mode).* **Green:** serous carcinoma samples. **Red:** non-serous carcinoma samples. Good separation between the studied groups/conditions was observed for both acquisition modes (ESI+/ESI-).

**A
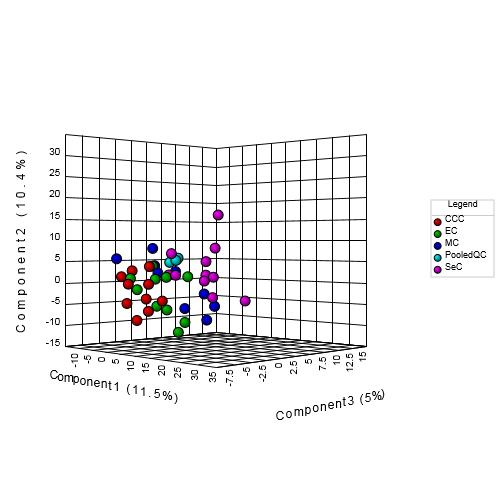
B**
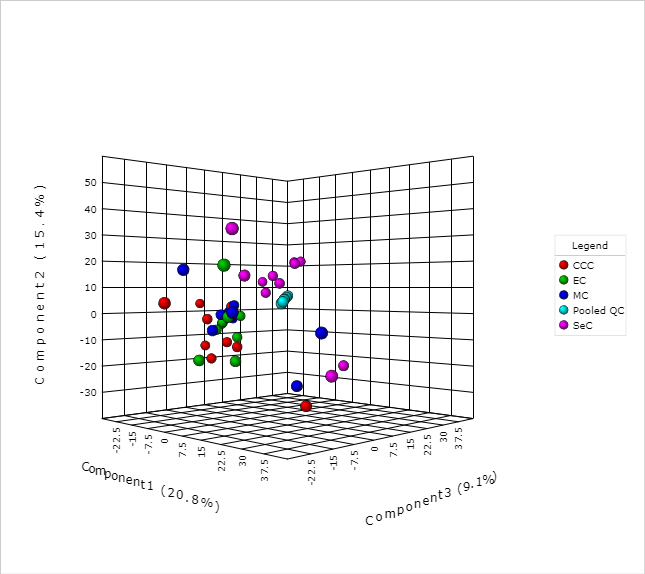


**ESI+**

**ESI-**

**Supplementary Fig. 5.** *Differences in serum metabolomic profiles in serous (SeC), endometrioid (EC), clear cell (CCC), and mucinous (MC) carcinoma samples observed in ESI+/HILIC and ESI-/HILIC mode*. The 3D-PLS-DA plots show comparisons of the various groups. As can be seen, the pooled QC samples (injected every 8 sample runs) cluster very tightly, which strongly suggests instrument stability throughout the LC-MS runs. The following data-filtering parameters were used for analysis: RSD of QC samples < 30%; average of each group over blanks ratios > 5; and total number of features = 540 (for ESI+ mode) and 147 (for ESI- mode). As with the other two chromatographic modes (lipidomic and metabolomic PFP-based runs), the structure of the data shows clear separation between the serous carcinoma and non-serous ovarian cancer samples—particularly between the SeC and EC/CCC groups.

**A
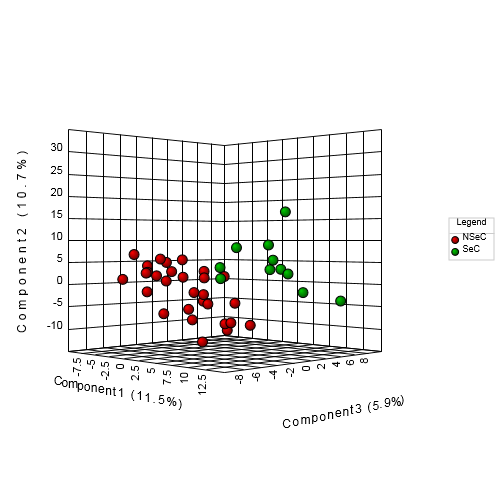
B
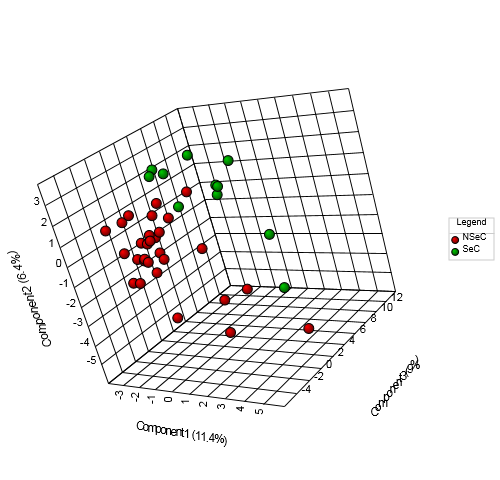
**

**ESI-**

**ESI+**

**Supplementary Fig. 6.** *Three-dimensional PLS-DA score plots for features detected in serous (SeC) or non-serous (NSeC) carcinoma samples (HILIC mode).* **Green:** serous carcinoma samples. **Red:** non-serous carcinoma samples. Good separation between the studied groups/conditions was observed for both acquisition modes (ESI+/ESI-).


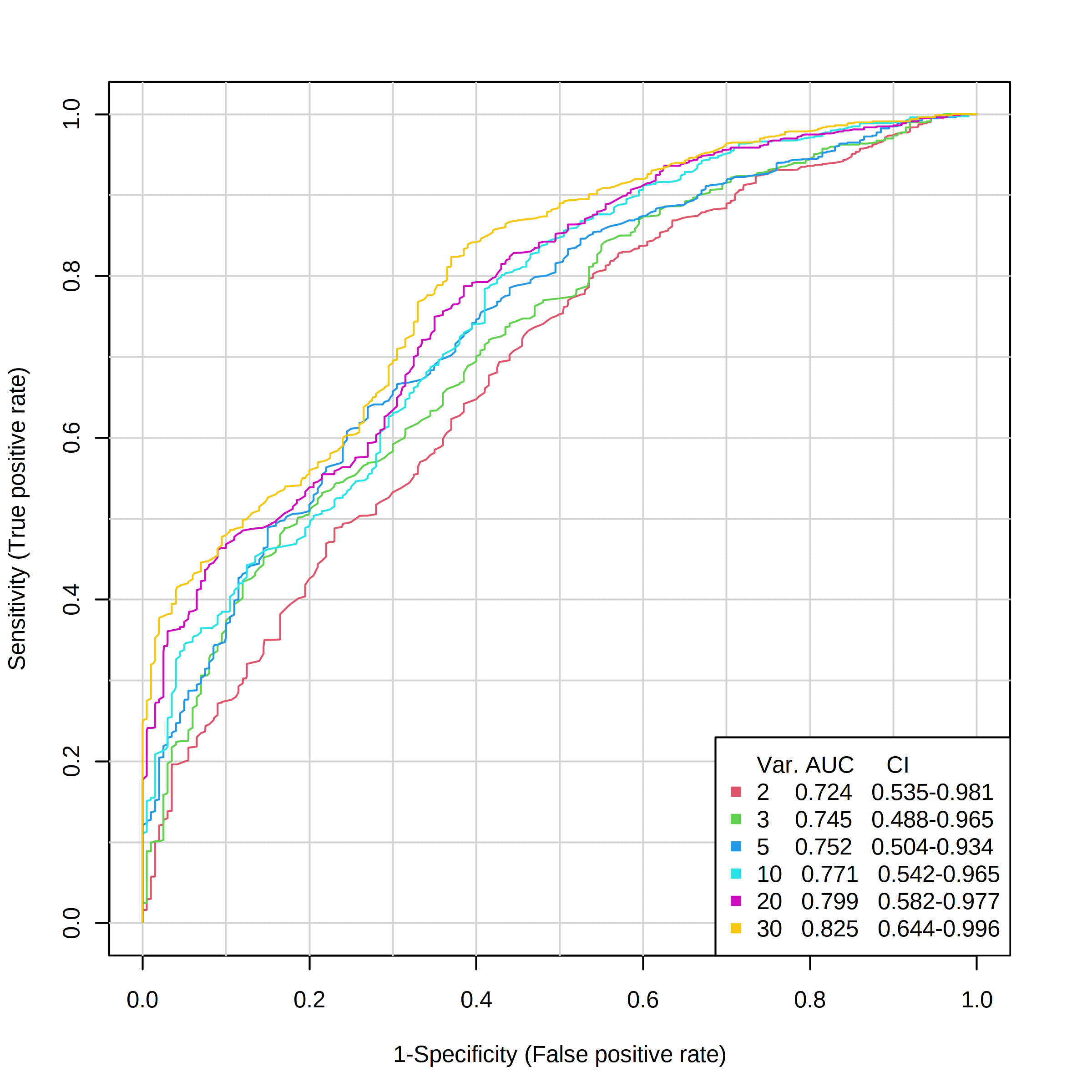


**Supplementary Fig. 7.** *Receiver-operating characteristic (ROC) curves for discriminating early-stage patients and advanced-stage ovarian cancer patients.* ROC curves of the 6 logistic models were generated using top 2-30 metabolomic markers.
